# Compared to placebo, long-term antibiotics resolve otitis media with effusion (OME) and prevent acute otitis media with perforation (AOMwiP) in a high-risk population: A randomized controlled trial

**DOI:** 10.1186/1471-2431-8-23

**Published:** 2008-06-02

**Authors:** Amanda J Leach, Peter S Morris, John D Mathews, the Chronic Otitis Media Intervention Trial – One (COMIT1) group

**Affiliations:** 1 Menzies School of Health Research, Charles Darwin University, Darwin, Northern Territory, Australia; 2 NT Clinical School, Darwin, Northern Territory, Australia; 3 Melbourne University, Melbourne, Australia

## Abstract

**Background:**

For children at high risk of chronic suppurative otitis media (CSOM), strategies to prevent acute otitis media with perforation (AOMwiP) may reduce progression to CSOM.

**Methods:**

In a double blind study in northern Australia, 103 Aboriginal infants with first detection of OME were randomised to receive either amoxicillin (50 mg/kg/d BD) or placebo for 24 weeks, or until bilateral aerated middle ears were diagnosed at two successive monthly examinations (success). Standardised clinical assessments and international standards for microbiology were used.

**Results:**

Five of 52 infants in the amoxicillin group and none of 51 infants in the placebo group achieved success at the end of therapy (Risk Difference = 9.6% [95% confidence interval 1.6,17.6]). Amoxicillin significantly reduced the proportion of children with i) perforation at the end of therapy (27% to 12% RD = -16% [-31,-1]), ii) recurrent perforation during therapy (18% to 4% RD = -14% [-25,-2]), and iii) reduced the proportion of examinations with a diagnosis of perforation during therapy (20% to 8% adjusted risk ratio 0.36 [0.15,0.83] p = 0.017). During therapy, the proportion of examinations with penicillin non-susceptible (MIC > 0.1 microg/ml) pneumococci was not significantly different between the amoxicillin group (34%) and the placebo group (40%). Beta-lactamase positive non-capsular *H. influenzae *(NCHi) were uncommon during therapy but more frequent in the amoxicillin group (10%) than placebo (5%).

**Conclusion:**

Aboriginal infants receiving continuous amoxicillin had more normal ears, fewer perforations, and less pneumococcal carriage. There was no statistically significant increase in resistant pneumococci or NCHi in amoxicillin children compared to placebo children who received regular paediatric care and antibiotic treatment for symptomatic illnesses.

## Background

Otitis media (OM) is an important health problem in young children. In most settings the pain or fever of acute OM (AOM) is of principal concern. However, in disadvantaged and Indigenous populations, where symptomatic AOM is less often reported, AOM with tympanic membrane perforation (AOMwiP) and progression to chronic suppurative OM (CSOM) can lead to hearing impairment and educational disadvantage. CSOM is estimated to currently affect between 65 and 330 million people, 60% of whom have a significant hearing loss [1] This global burden mainly affects South East Asia, Western Pacific, Africa and ethnic minorities. A 2001 cross-sectional survey of Aboriginal children aged 6 months to 2.5 years living in 29 remote Aboriginal communities in the Northern Territory of Australia, showed perforation rates varying from 0% to 60% (mean rate 24%) between communities [2]. In longitudinal studies of Aboriginal children, OM has been shown to commence within weeks of birth, shortly after nasopharyngeal colonisation with *Streptococcus pneumoniae*, non-capsular *Haemophilus influenzae *(NCHi) and *Moraxella catarrhalis*.[3] This link between bacterial colonisation and early onset of persistent OM suggested to us that long-term antibiotic therapy could be of benefit for Aboriginal infants and others in high-risk populations. The impact of antibiotics has been unclear in populations where rates of CSOM exceed 4%, although systematic reviews demonstrate that antibiotics do prevent some episodes of AOM in more affluent populations, [4,5] and are particularly beneficial for children with more severe OM [6].

## Methods

### Aims

To compare the effects of long-term antibiotics and placebo on otitis media with effusion (OME) and nasopharyngeal carriage of antibiotic-resistant OM bacterial pathogens in a high-risk population.

### Participants and Setting

The study was conducted in three Aboriginal communities located 70 Km from Darwin, in tropical northern Australia. During the study period breastfeeding was universal, parental smoking extremely common, and formal childcare was unavailable. Data collection commenced in 1996 and was completed in 2001, when pneumococcal conjugate vaccine was about to be licensed in Australia.

### Eligibility and exclusion criteria

Enrolled infants were Aboriginal, less than 12 months of age and resident in a study community. Infants were enrolled as soon as possible after birth, examined every two weeks, and those with unilateral or bilateral otitis media with effusion (OME) were eligible for randomization. Infants were excluded on the basis of prematurity (<34 weeks), chronic infection requiring prophylactic antibiotic therapy, craniofacial abnormalities or immune deficiency syndromes.

### Ethical and Funding Issues

The study was approved by the Top End Human Research Ethics Committee and Aboriginal Sub-Committee, and the Tiwi Health Board. Funding was provided by the National Health and Medical Research Council, which had no involvement in study design, management, or reporting of results.

### Consent procedures

Written informed consent was sought before enrolment and also before randomization; families were given at least 2 weeks to consider consent. Initially, consent for microbiological assessments prior to randomisation was not requested.

### Randomised Intervention

Randomized participants received either amoxicillin (50 mg/kg/day bid) or placebo equivalent volume for 24 weeks or until bilateral normal middle ear status was detected at two consecutive monthly examinations (success). Block randomisation was stratified by age. Allocation was concealed from children, family, investigators, and those providing routine clinical care. Blinding was achieved by using a placebo similar in packaging, colour, consistency and smell to amoxicillin suspension. Intercurrent illnesses were managed according to local community treatment guidelines [7].

Further details of randomisation [see Additional File 1].

### Diagnosis and Outcome assessments

#### Eligibility and scheduling

Baseline characteristics and pre-enrolment ear disease were assessed from clinical records. Infants were examined fortnightly from enrolment to randomization, and monthly over the 24 week intervention period or until success was documented.

#### Assessments

At each study visit a paediatrician or pediatric nurse trained by an Australian expert used video pneumatic otoscopy and tympanometry (Grason Stadler GSI 38) to assess ear status. Clinic notes were reviewed to assess inter-current illness and side effects. Nasopharyngeal swabs were collected at baseline and at each post-randomisation visit, and processed as previously reported to detect pneumococci, *Haemophilus influenzae *and *Moraxella catarrhalis *[8-10].

#### Criteria for otitis media categories (Table 1)

**Table 1:** Diagnostic criteria and severity scale for worse ear

**Diagnosis**	**Definition**	**Severity scale for worse ear**.
Normal	Absence of middle ear inflammation or infection, normal mobility on pneumatic otoscopy, and type A, C1 or C2 tympanogram.	0
Otitis media with effusion (OME)	Fluid behind an intact tympanic membrane (TM), reduced mobility on pneumatic otoscopy or type B tympanogram, with or without mild bulging, and in the absence of signs or symptoms of acute infection.	1
Acute Otitis Media without perforation (AOMwoP)	Fluid behind an intact TM, reduced mobility on pneumatic otoscopy or type B tympanogram, with moderate or marked bulging, with or without symptoms of acute infection and without signs of recent perforation.	2
AOM with perforation (AOMwiP)	Discharge through a perforated TM for less than 6 weeks.	3
Chronic suppurative otitis media (CSOM)	Discharge through a perforated TM for more than 6 weeks despite appropriate treatment for AOMwiP.	4

Diagnostic criteria were as previously reported [11]. Table 1 gives definitions and the severity scale used to assign the worse (highest score) ear diagnosis at each examination. In this study, ear examinations that demonstrated type B tympanogram, reduced mobility on pneumatic otoscopy and a marked or moderately bulging TM were categorised as AOM without perforation (AOMwoP) even if the child was asymptomatic.

#### Success

The primary outcome ('success'), was detection of bilateral aerated middle ears (confirmed with tympanometry) at two consecutive monthly visits.

### Statistical power and methods

We aimed to achieve a sample size of 118 that would provide 80% power to detect a 20% increase in success rate (5% to 25%). This would also give 88% power to detect a 30% reduction in perforation at the end of therapy (60% to 30%). Interim analysis and stopping rule was intended to be applied if the success rate in the active arm was 40%.

Data were analysed according to a pre-determined plan. We used the outcome in the worse ear (the ear with the highest severity score) to avoid dependence between ears. Estimates of treatment effect are given as the difference between amoxicillin and placebo groups in the prevalence of each outcome at the end of therapy, as a difference in the proportion of examinations during therapy (risk difference and unadjusted 95% confidence interval) or as a adjusted risk ratio and 95% confidence interval (adjusted for repeat child examinations using generalised linear models – Poisson). For children who failed to complete therapy, the last available assessment was used. Two sided significance tests were used throughout. All calculations used Stata version 9 [12].

## Results

### Participants [see Additional File 1]

**Figure 1:**
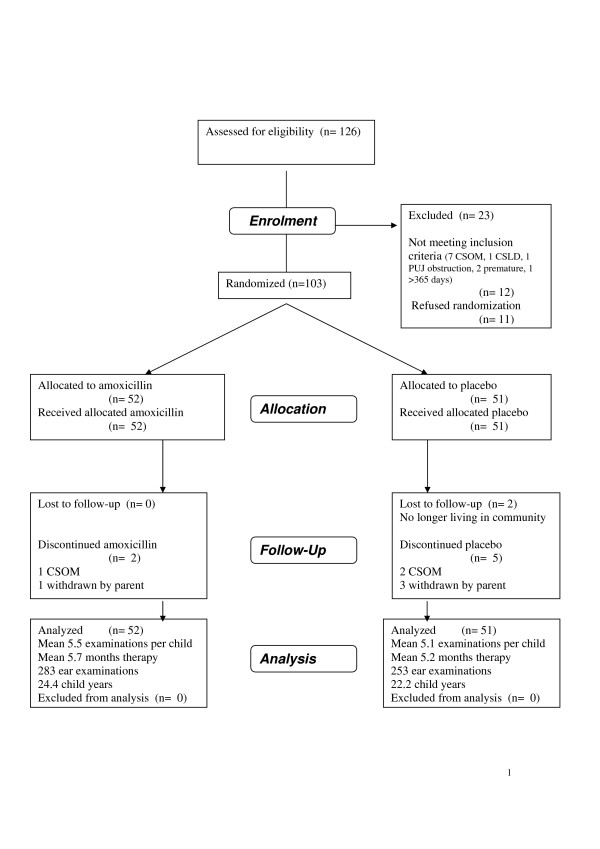
**The Consort E-Flowchart.** Participant Flow in the COMIT1 Trial.

From 188 births in the study period, family consent was obtained to enrol 126 infants. 103 were randomised (Figure 1). Details of non-randomised infants and of those who discontinued amoxicillin (n = 2) or placebo (n = 7).

### Pre-randomisation details for randomised children (Table 2)

**Table 2:** Baseline characteristics and ear assessments between enrolment and randomisation (including day of enrolment and day of randomisation)

	**Amoxicillin**	**Placebo**
	**N = 52**	**N = 51**
Mean age (years) of mothers	24.5 (N = 52)	23.3 (N = 50)
Mean gestation (weeks)	38.3 (N = 43)	38.5 (N = 46)
Mean birth weight (gm)	2812 (N = 51)	3155 (N = 50)
Male	24 (46%)	30 (59%)
**Enrolment to randomisation (inclusive)**		
Mean age (mo) at enrolment	4.2	3.2
Mean duration to randomisation (months)	1.6	1.8
Number of study visits	155	166
Mean visits per child	2.5	2.6
**Number (%) of examinations with the following worst ear status(enrolment to randomisation, inclusive)**		
	N = 155	N = 166
Normal	18 (12%)	19 (11%)
Otitis media with effusion (OME)	83 (54%)	82 (49%)
Acute Otitis Media (AOM) without perforation (AOMwoP)	41 (26%)	44 (26%)
AOM with perforation (AOMwiP)	6 (4%)	10 (6%)
Dry perforation	1	1
Chronic Suppurative Otitis Media (CSOM)	0	2
Any suppurative OM†	47 (30%)	56 (34%)
Any perforation‡	7 (5%)	13 (8%)

≸AOMwoP, AOMwiP or CSOM;

≹AOMwiP, dry perforation or CSOM

In the period from birth to randomisation, diagnoses of OM or respiratory illnesses other than OM, and antibiotic use (oral or IM) had been documented by clinic staff for more than half the children in each group (data not shown). All children were less than 12 months of age at enrolment. There were 321 examinations by study staff between enrolment and randomisation; bilateral normal ears were diagnosed in 12% of examinations, OME in about 50%, AOM in 26%, and perforations in less than 10%.

52 infants were allocated to amoxicillin and 51 to placebo, and the groups were similar for mean age of mothers, mean infant gestational age, proportion male, number of study visits before randomisation, age at randomisation, and distribution of ear-states prior to randomisation. Mean birth weight was significantly greater for placebo infants (p = 0.0013).

### Ear states at randomisation

At randomisation 50 (96%) of amoxicillin infants and 49 (96%) of placebo infants had OME as the worst ear diagnosis; 3 infants had resolving AOM and started the intervention study after an additional week of antibiotic treatment; one infant (placebo) had unilateral dry perforation.

### Nasopharyngeal carriage at randomisation [see Additional File 2]

On the day of randomisation, carriage of each respiratory bacterial pathogen was high in both groups (between 73% and 81%). All three OM bacterial pathogens were carried by half of the children in each group (54% and 53% respectively); penicillin non-susceptible (MIC> 0.1 μg.ml) pneumococci were carried by 27% and 37% in amoxicillin and placebo groups, respectively. Beta-lactamase producing *H. influenzae *were detected in 6% and 2% of children respectively. None of these differences reached statistical significance.

### Outcomes at the end of therapy (Table 3)

**Table 3:** Ear assessments and nasopharyngeal carriage at the end of therapy

	**Amoxicillin**	**Placebo**	
	**n = 52**	**n = 51**	
Mean duration of therapy (months)	5.7	5.2	
Mean age (months) at the end of therapy	11.5	10.2	
**Number of children (%) with the following worst ear status at the end of therapy**			
	**Amoxicillin**	**Placebo**	**Risk Difference**
	**n = 52**	**n = 51**	**[95% CI]**
Success (bilateral normal ears at 2 successive monthly visits)	5 (9.6%)	0	+9.6% [1.6, 17.6]
Normal	6 (12%)	0	+12% [3, 20]
OME	28 (54%)	26 (51%)	+3% [-16, 22]
AOM without perforation	12 (23%)	11 (22%)	+1.5% [-15, 18]
AOM with perforation	5 (10%)	11 (22%)	-12% [-26, 2]
Dry perforation	0	1 (2%)	-2% [-6, 2]
CSOM	1 (2%)	2 (4%)	-2% [-9, 5]
Any suppurative OM†	18 (35%)	24 (47%)	-12% [-31, 6]
Any perforation‡	6 (12%)	14 (27%)	-16% [-31, -1]
Any active perforation§	6 (12%)	13 (26%)	-14% [-29, 1]
Bilateral AOM without perforation	4 (8%)	7 (13%)	+5% [-6, 17]
Bilateral any perforation‡	1 (2%)	7 (14%)	-12% [-22, -2]
**Number of children (%) with nasopharyngeal carriage of the following OM pathogens^¥ ^at end of therapy**			
	**Amoxicillin**	**Placebo**	**Risk Difference**
	**n = 50**	**n = 47**	**[95% CI]**
*Streptococcus pneumoniae*	29 (58%)	33 (70%)	-12% [-31, 7]
non-capsular *Haemophilus influenzae*	36 (72%)	29 (62%)	+10% [-8, 29]
*Moraxella catarrhalis*	44 (88%)	43 (91%)	-3% [-16,9]
All Spn, NCHi and M.cat	23 (46%)	21 (45%)	1% [-19, 21]
Penicillin non-susceptible Spn‡	17 (34%)	19 (40%)	-6% [-26, 13]
Penicillin resistant Spn††	1 (2%)	2 (4%)	-2.3% [-9, 5]
NCHi beta-lactamase positive	6 (12%)	1 (2%)	+10% [-0.03, 20]

≸AOM, AOMwiP or CSOM; ‡ AOMwiP, dry perforation or CSOM; §AOMwiP or CSOM; ¥ Spn *Streptococcus pneumoniae*. NCHi non-capsular *Haemophilus influenzae*. Mcat *Moraxella catarrhalis*. ‡Penicillin intermediate or resistant Spn (MIC > = 0.1 μg/ml) ††Penicillin resistant Spn (MIC > 1.0 μg/ml)

No infant was withdrawn as a result of direct adverse reaction due to medication. The mean duration of therapy was 5.7 months for amoxicillin infants and 5.2 months for placebo infants. Mean ages at the end of therapy were 11.5 and 10.2 months.

There were 5 successes (bilateral aerated middle ears at two successive monthly examinations) in the amoxicillin group and none in the placebo group (Risk Difference = +9.6% [95% Confidence Interval 1.6, 17.6]). Six infants in the amoxicillin group had bilateral normal ears at the end of therapy compared to no infant in the placebo group (RD = +12% [3,20]). Most children had OME (54% and 51%, respectively); AOMwoP was diagnosed in 23% and 22% children. Perforation was significantly reduced in the amoxicillin group (12%) compared with placebo (27%).

### Ear Assessments during therapy [see Additional Files 3 and 4]

Bilaterally normal ears were seen at least once in 11 (21%) amoxicillin infants and 5 (10%) placebo infants. More children in the amoxicillin group had at least one diagnosis of OME and more had AOMwoP. Fewer amoxicillin children had AOMwiP detected (25% and 33%, respectively), and recurrent AOMwiP was identified in significantly fewer amoxicillin children (4%) and than placebo children (18%) (RD = -14% [-25, -2]).

Results were similar when expressed as a proportion of examinations for each group. Bilateral normal ears were detected in 6% and 2% of visits in amoxicillin and placebo infants, respectively (adjusted risk ratio = 2.7 [0.78, 9.13] p = 0.116). OME was the worst ear diagnosis at about half the visits in each group, AOMwoP was detected in about one quarter of visits. AOMwiP was less often diagnosed in children receiving amoxicillin (8%) than placebo (19%) (adjusted risk ratio = 0.36 [0.16, 0.84] p = 0.017). For combined diagnostic categories of any suppurative OM, any perforation, and any active perforation, significant differences in perforation were found. Antibiotic prescribing for a clinical indication was less often required at examinations of amoxicillin children (55%) then placebo children (68%) (adjusted risk ratio = 0.67 [0.36, 1.22] p = 0.190). Tables S2a and S2b give more detail of ear status over the course of the trial [see Additional files 3 and 4].

### Nasopharyngeal outcomes at the end of therapy (Table 2)

At the end of therapy, carriage of OM pathogens, and carriage of penicillin non-susceptible S. pneumoniae (34% in amoxicillin group and 40% in placebo group) was not significantly different between groups.

### Nasopharyngeal outcomes during therapy [see Additional Files 3 and 4]

Over the course of the RCT, fewer amoxicillin swabs (59%) than placebo swabs (78%) were positive for pneumococcus (adjusted risk ratio = 0.77 [0.67, 0.88] p = 0.000). Recovery of NCHi was ~70% in each group. There were non-significant differences in 7-valent pneumococcal conjugate vaccine serotypes (42% versus 48% in amoxicillin and placebo swabs, respectively, data not shown) and *M. catarrhalis *(85% versus 90%). Fewer amoxicillin swabs cultured all 3 pathogens simultaneously (46% versus 58%), but this was not statistically significant (adjusted risk ratio = 0.64 [0.34, 1.23] p = 0.183). During therapy, children in the amoxicillin group also carried fewer penicillin non-susceptible pneumococci (34% versus 40% in amoxicillin and placebo, respectively), and fewer macrolide non-susceptible (16% versus 20%, data not shown) and multi-drug resistant (15% versus 20%, data not shown) pneumococci. Beta-lactamase producing NCHi were detected more often in amoxicillin (10%) than placebo (5%) swabs; these latter differences did not reach statistical significance. Tables S2b and S3b give more detail of carriage status over the course of the trial [see Additional Files 3 and 4].

## Discussion

We have studied OM in Aboriginal communities where almost all children have persistent middle ear disease from an early age [3]; nevertheless, our results could also be relevant for other high-risk groups. The primary outcome from our double-blind RCT is that amoxicillin significantly increased the proportion of children achieving normal middle ears (bilaterally aerated) at consecutive monthly examinations. Amoxicillin also reduced the proportion of children with any perforation. The proportion with any suppurative OM was also less in the amoxicillin group at the end of therapy, but not significantly so (Table 2). The proportion of children on amoxicillin experiencing at least two episodes of AOMwiP during therapy was less than for placebo children (4% versus 18%, respectively). There was no difference between placebo and amoxicillin in the proportion of children with OME (~52% in each group) or AOMwoP (~22%) at the end of therapy. Risk differences during therapy (supplementary material) were consistent with the end of therapy findings. Pneumococcal non-susceptibility was similar in amoxicillin and placebo groups, however a greater proportion of children in the amoxicillin group were colonised by beta-lactamase producing *H. influenzae *at both baseline and at end of therapy.

Our findings can be understood in terms of the natural history of OM in high-risk populations. In Aboriginal children, AOM is usually asymptomatic with parents unaware that their child is unwell. In community-based screening, [13] some 20% to 30% of Aboriginal children have bulging eardrums. Only a minority have the typical symptoms (sudden onset of middle ear effusion with pain or fever) which define AOM in low-risk populations [14]. To explain our findings in this high-risk population, we suggest that in placebo recipients, asymptomatic middle ear infection often progresses to AOM with perforation, whereas continuous amoxicillin helps to prevent that progression. This is consistent with our previous study of short-term antibiotics for asymptomatic AOM which indicated that the majority of children still satisfy the criteria for AOM despite treatment for 7 to 14 days. [11] The present study suggests that therapy can be more successful if it is maintained long-term. We believe that this can be explained by the association of bacterial load with increased severity of OM [15]. It may be that continuous antibiotics maintain bacterial load below a critical threshold for more severe disease.

Our trial supports the notion that asymptomatic bulging of the tympanic membrane is an important predictor of AOM and perforation [16]; this has not been emphasised in the AAP and AAFP guideline [14]. Furthermore, the implications of withholding antibiotic therapy in children with bulging eardrums have not previously been evaluated in high quality studies. Guidelines for management of AOM in Indigenous children define AOM as middle ear effusion with either bulging of the tympanic membrane, recent discharge, ear pain, or redness [17]. There is no option to withhold antibiotics in an Aboriginal child with AOM; for children in whom AOM is associated with perforation, longer courses of antibiotics are recommended. Our RCT supports that recommendation.

A recent meta-analysis of individual patient data from studies in low-risk populations identified subgroups most likely to benefit from antibiotic therapy [6]. In children younger than 2 years of age with bilateral acute otitis media, 55% of controls and 30% on antibiotics still had pain, fever, or both at 3–7 days, with a rate difference between these groups of -25% (95% CI -36% to -14%), resulting in a number-needed-to-treat (NNT) of four children. In children with otorrhoea the rate difference and NNT, respectively, were -36% (-53% to -19%) and three, whereas in children without otorrhoea the equivalent values were -14% (-23% to -5%) and eight. From our results in Aboriginal children (Table 2), the NNT would be about ten for normalisation of ears, about six for prevention of perforation, and about four for either beneficial outcome. If compliance could be guaranteed in Aboriginal children, each NNT would likely be reduced.

What are the microbiological implications of long-term amoxicillin? Our study shows a significant reduction in pneumococcal carriage during amoxicillin therapy. Despite the positive results from our RCT it is unclear whether withholding antibiotics in an asymptomatic child at risk of perforation will result in a clinical deterioration that outweighs the theoretical risk of increasing antibiotic resistance should the child be treated. It is reassuring that in our RCT there was no increase in the carriage of penicillin-resistant pneumococci as a result of treatment, nor any difference in antibiotics prescribed for intercurrent illnesses between amoxicillin and placebo children. Indeed there was a tendency for *fewer *children in the amoxicillin group to be colonised by penicillin-resistant pneumococci. On the other hand, although beta-lactamase producing *H. influenzae *were uncommon in both groups, they were detected more frequently in children receiving amoxicillin; however, as this non-significant trend was also present before the start of trial therapy, it could be a chance finding.

Empirical studies of resistance are not available from other randomised controlled trials of long term antibiotics for otitis media [18]. The study of perforation in Alaska natives [19], which also found that long-term antibiotics were beneficial, did not report on antibiotic resistance. We note that by 1997, 25% of Australian clinical isolates were penicillin non-susceptible, and high levels of penicillin resistance were reported from Aboriginal communities from the mid 1980s [20].

We have previously shown that OM is more advanced when multiple pathogens colonise the nasopharynx simultaneously and at high density [15] We suggest that the effect of long-term amoxicillin is to reduce the density and multiplicity of bacterial carriage by reducing the rate of acquisition of new strains and/or the rate of proliferation of those already acquired. In the Finnish OM study [21] 28% of new pneumococcal acquisitions were associated with pneumococcal AOM, compared with only 11% for established carriage.

We believe that our study of long-term antibiotic use is very relevant for Aboriginal and other populations at high risk of tympanic membrane perforation. Unfortunately conjugate pneumococcal vaccine has not significantly reduced ear disease in Australian Aboriginal children. A recent pediatric outreach program in Far North Queensland confirms that CSOM is the most prevalent health problem in Aboriginal children [22]. The poor school achievement of Aboriginal children, already disadvantaged by poverty, has been partially attributed to the chronic hearing loss [23].

Our study shows that Aboriginal infants with OME receiving continuous amoxicillin had fewer perforations, more normal ears, less pneumococcal carriage and fewer resistant pneumococci than children who received regular paediatric care and antibiotic treatment for acute illnesses but without continuous amoxicillin. The option to use long-term antibiotics would be less relevant for western populations where the outcome of untreated OM is less severe, and the balance of benefit and risk is less influenced by the risk of a severe clinical outcome, and more influenced by the potential for increased antibiotic resistance in the child and the community.

## Conclusion

Our study shows that Aboriginal infants with OME receiving continuous amoxicillin had more normal ears, fewer perforations and less pneumococcal carriage. There was no statistically significant increase in resistant pneumococci or NCHi in amoxicillin children compared to placebo children who received regular paediatric care and antibiotic treatment for symptomatic illnesses. The option to use long-term antibiotics would be less relevant for western populations where the outcome of untreated OM is less severe, and the balance of benefit and risk is less influenced by the risk of a severe clinical outcome, and more influenced by the potential for increased antibiotic resistance in the child and the community.

## Competing interests

The authors declare that they have no competing interests.

## Authors' contributions

AJL, PSM and JDM participated in the design of the study, secured funding and performed statistical analyses. PSM and AJL conducted and supervised data collection. PSM performed and supervised clinical assessments. AJL performed and supervised laboratory analyses. All authors contributed to the manuscript and approved the final version.

## Pre-publication history

The pre-publication history for this paper can be accessed here:



## Supplementary Material

Additional file 1 **Randomisation details and characteristics of non-randomised and discontinued children**. Further details of method of randomisation (e.g. allocation concealment and blinding), reasons for ineligibility to be randomised, and withdrawals. Click here for file

Additional file 2 **Table 1. Carriage at randomisation**. Nasopharyngeal carriage on the day of randomisation. Click here for file

Additional file 3 **Tables 2a & 2b. Ear assessments and carriage during therapy**. Table 2a provides number (%) of children with each worst ear status at least once during therapy, and number of examinations (%) with each worst ear status during therapy. Table 2b provides number of swabs (%) with each OM pathogen during therapy. Click here for file

Additional file 4 **Tables 3a & 3b. Incidence rate ratios**. Discussion of how incidence rate ratio was applied to data from this high-risk population. Table 3a Incidence per child year and incidence rate ratio (IRR) of each worst ear status during therapy. Table 3b: Incidence per child year and incidence rate ratio (IRR) of nasopharyngeal carriage of each OM pathogen^¥ ^during therapy. Click here for file
