# Supplementary material for: Compared to placebo, long-term antibiotics resolve otitis media with effusion (OME) and prevent acute otitis media with perforation (AOMwiP) in a high-risk population: A randomized controlled trial
Source: BMC Pediatr. 2008 Jun 2;8:23. doi: 10.1186/1471-2431-8-23 (PMC2443129; doi:10.1186/1471-2431-8-23)
Supplement: Additional file 1 — Randomisation details and characteristics of non-randomised and discontinued children. Further details of method of randomisation (e.g. allocation concealment and blinding), reasons for ineligibility to be randomised, and withdrawals. [file 1471-2431-8-23-S1.doc]

**ADDITIONAL FILE 1: Randomisation details and characteristics of non-randomised and discontinued children.**

**Randomization details**

## Sequence generation : A computer generated random number series was stratified by age at randomization (less than 6 months versus greater than 6 months) and allocation was randomized within blocks of 7 subjects.

**Allocation concealment :** Allocation to placebo or amoxicillin, and the use and size of block randomization was concealed from investigators until data collection was completed.

**Implementation :** Participants were enrolled by project staff and eligibility for randomization was determined by investigators. Participants were consecutively allocated a random number according to the sequence provided by the systems manager.

**Blinding :** Placebo was designed, manufactured and packaged by Institute of Drug Technology, Melbourne. Bottles were provided by the manufacturers of amoxicillin. Original amoxicillin labels were removed before applying the study label. Senior staff at the community clinics had access to the allocation via a locked box containing stapled double envelopes. Clinic staff were not involved in data collection. The biostatistician was provided with the codes and the allocation to either ‘A’ or ‘B’, but was unaware of whether ‘A’ or ‘B’ was amoxicillin or placebo.

**Compliance with study intervention :** Medication was prepared each Monday by a research officer. Supervised twice daily medication delivery was provided by local residents if requested by the mother. Residual volumes were measured by independent laboratory staff.

**Participants**

**Non-randomised children**

Twenty three enrolled children could not be randomised; twelve eventually met exclusion criteria (see participant flow diagram), 11 agreed to ear examinations but refused consent for randomisation. Eight (40%) of the 20 age-eligible non-randomised infants were male. The mean age at enrolment was 4.9 months. Sixty six of 68 ear examinations in the first year of life of these 20 non-randomised children were successful; 9 diagnoses (14%) in 2 children were normal, 11 (17%) in 8 children were OME, 19 (29%) in 11 children were AOMwoP; 18 (27%) in 8 children were AOMwiP, and 9 (14%) in 5 children were CSOM.

**Discontinued**

Two children in the amoxicillin group (1 CSOM, 1 parent refusal) and seven in the placebo group (2 CSOM, 3 parent refusals and 2 moved from the community) failed to complete therapy. All collected data were analysed (see Figure 1).
