# Supplementary material for: Compared to placebo, long-term antibiotics resolve otitis media with effusion (OME) and prevent acute otitis media with perforation (AOMwiP) in a high-risk population: A randomized controlled trial
Source: BMC Pediatr. 2008 Jun 2;8:23. doi: 10.1186/1471-2431-8-23 (PMC2443129; doi:10.1186/1471-2431-8-23)
Supplement: Additional file 2 — Table 1. Carriage at randomisation. Nasopharyngeal carriage on the day of randomisation. [file 1471-2431-8-23-S2.doc]

**ADDITIONAL FILE 2: Supplementary results table 1. Carriage at randomisation.**

**Table S1. Nasopharyngeal carriage on the day of randomisation**

|  | **Amoxycillin**  **N=52** | **Placebo**  **N=51** |
| --- | --- | --- |
| **Number of children swabbed at randomisation** | **52** | **51** |
| **Number (%) of children with nasopharyngeal carriage of the following** **OM pathogens**^¥^  **at randomisation** | | |
| 1. Spn | 42  (81%) | 40  (78%) |
| 1. NCHi | 38  (73%) | 41  (80%) |
| 1. Mcat | 40  (77%) | 40  (78%) |
| 1. All Spn and NCHi and Mcat | 28  (54%) | 27  (53%) |
| 1. Penicillin intermediate or high level resistant Spn (MIC > 0.1μg/ml) | 14  (27%) | 19  (37%) |
| 1. NCHi beta-lactamase positive | 3  (6%) | 1  (2%) |

^¥^ Spn *Streptococcus pneumoniae*. NCHi non-capsular *Haemophilus influenzae*. M. cat *Moraxella catarrhalis.*
