# Supplementary material for: Compared to placebo, long-term antibiotics resolve otitis media with effusion (OME) and prevent acute otitis media with perforation (AOMwiP) in a high-risk population: A randomized controlled trial
Source: BMC Pediatr. 2008 Jun 2;8:23. doi: 10.1186/1471-2431-8-23 (PMC2443129; doi:10.1186/1471-2431-8-23)
Supplement: Additional file 3 — Tables 2a & 2b. Ear assessments and carriage during therapy. Table 2a provides number (%) of children with each worst ear status at least once during therapy, and number of examinations (%) with each worst ear status during therapy. Table 2b provides number of swabs (%) with each OM pathogen during therapy. [file 1471-2431-8-23-S3.doc]

**ADDITIONAL FILE 3: Supplementary results tables 2a & 2b. Ear assessments and carriage during therapy.**

| **Table S2a: Ear assessments during therapy** (excluding day of randomisation) | | | |  |
| --- | --- | --- | --- | --- |
|  | **Amoxicillin** | **Placebo** |  |  |
|  | **n=52** | **n=51** |  |  |
| Child years of therapy | 24.4 | 22.2 |  |  |
| Mean study visits per child | 5.5 | 5.1 |  |  |
| Number of study visits | 283 | 253 |  |  |
| **Number (%) of children with the following worst ear status at least once during therapy** | | | |  |
|  | **Amoxicillin** | **Placebo** | **Risk Difference** |  |
|  | **n=52** | **n=51** | **[95% CI]** |  |
| Normal | 11 (21%) | 5 (10%) | +11% [-2, 25] |  |
| OME | 49 (94%) | 44 (86%) | +8% [-3, 19] |  |
| AOM without perforation | 37 (71%) | 28 (55%) | +16% [-2, 35] |  |
| AOM with perforation | 13 (25%) | 17 (33%) | -8% [-26, 9] |  |
| Dry perforation | 1 (2%) | 2 (4%) | -2% [-9, 5] |  |
| CSOM | 1 (2%) | 3 (6%) | -4% [-11, 3] |  |
| Any suppurative OM† | 40 (79%) | 37 (75%) | +4% [-12, 21] |  |
| Any perforation‡ | 13 (25%) | 17 (33%) | -8% [-26,9] |  |
| Any active perforation§ | 13 (25%) | 17 (33%) | -8% [-26, 9] |  |
| Any recurrent AOMwoP (>2 episodes) | 12 (23%) | 9 (18%) | +5% [-10, 21] |  |
| Any recurrent AOMwiP (>2 episodes) | 2 (4%) | 9 (18%) | -14% [-25, -2] |  |
| **Number of examinations (%) with the following worst ear status during therapy** | | | |  |
|  | **Amoxicillin** | **Placebo** | **Risk Ratio** | **P**** |
|  | **N=283** | **N=253** | **[95% CI]** | **adjusted** |
|  |  |  | **Adjusted Poisson** |  |
| Normal | 18(6%) | 5 (2%) | 2.7[0.78, 9.13] | 0.116 |
| OME | 161(57%) | 132 (52%) | 0.91[0.46, 1.78] | 0.776 |
| AOM without perforation | 79(28%) | 61(24%) | 0.96[0.48, 1.95] | 0.917 |
| AOM with perforation | 23(8%) | 48(19%) | 0.36[0.16, 0.84] | 0.017 |
| Dry perforation | 1(0.4%) | 4(1.6%) | 0.19[0.01, 2.46] | 0.202 |
| CSOM | 1 (0.4%) | 3(1.1%) | 0.25[0.02, 2.65] | 0.248 |
| Any suppurative OM† | 103(36%) | 112(44%) | 0.69[0.37, 1.28] | 0.239 |
| Any perforation‡ | 25(9%) | 55(22%) | 0.34[0.15, 0.81] | 0.015 |
| Any active perforation§ | 24(8%) | 52(20%) | 0.36[0.15, 0.83] | 0.017 |
| Antibiotics prescribed¥ | 156(55%) | 175(68%) | 0.67[0.36, 1.22] | 0.190 |

†AOM, AOMwiP or CSOM;

‡ AOMwiP, dry perforation or CSOM;

§ AOMwiP or CSOM;

¥ Number of visits at which clinically indicated antibiotics were prescribed or had been prescribed in previous month

**Adjusted for repeated examinations in the same child.

| **Table S2b: Nasopharyngeal carriage during therapy.** | | | |  |
| --- | --- | --- | --- | --- |
| **Number of swabs (%) with the following OM pathogens**^¥^ **during therapy** | | | |  |
|  | **Amoxicillin** | **Placebo** | **Risk Ratio** | **P**** |
|  | **N=283** | **N=251** | **[95% CI]** | **adjusted** |
|  |  |  | **Adjusted Poisson** |  |
| Spn | 167 (59%) | 196 (78%) | 0.77[0.67, 0.88] | 0.000 |
| NCHi | 202 (71%) | 176 (70%) | 0.86[0.47, 1.6] | 0.615 |
| M.cat | 241 (85%) | 225 (90%) | 0.80[0.44, 1.46] | 0.46 |
| All Spn, NCHi and M.cat | 130 (46%) | 146 (58%) | 0.64[0.34, 1.23] | 0.183 |
| Penicillin non-susceptible Spn‡ | 95 (34%) | 100 (40%) | 0.70[0.37, 1.3] | 0.276 |
| Beta-lactamase producing NCHi | 28 (10%) | 13 (5%) | 1.6[0.63, 4.1] | 0.321 |

^¥^ Spn *Streptococcus pneumoniae*. NCHi non-capsular *Haemophilus influenzae*. M. cat *Moraxella catarrhalis.*

‡Penicillin intermediate or high level resistant Spn (MIC >= 0.1μg/ml)

**Adjusted for repeated examinations in the same child.
