# Supplementary material for: Compared to placebo, long-term antibiotics resolve otitis media with effusion (OME) and prevent acute otitis media with perforation (AOMwiP) in a high-risk population: A randomized controlled trial
Source: BMC Pediatr. 2008 Jun 2;8:23. doi: 10.1186/1471-2431-8-23 (PMC2443129; doi:10.1186/1471-2431-8-23)
Supplement: Additional file 4 — Tables 3a & 3b. Incidence rate ratios. Discussion of how incidence rate ratio was applied to data from this high-risk population. Table 3a Incidence per child year and incidence rate ratio (IRR) of each worst ear status during therapy. Table 3b: Incidence per child year and incidence rate ratio (IRR) of nasopharyngeal carriage of each OM pathogen¥ during therapy. [file 1471-2431-8-23-S4.doc]

**ADDITIONAL FILE 4: Supplementary results tables 3a & 3b. Incidence rate ratios.**

In this high risk population, OM diagnoses which were made at scheduled monthly visits could not be assumed to be new infections because AOM is usually asymptomatic and the signs of acute infection (bulging TM, perforation or discharge) tend to persist. To enable comparisons with similar studies in other populations, we report the rate of detection of AOM during the intervention period. We cannot assume that a previously abnormal state had resolved in the intervening period (usually 4 weeks) since the previous examination (as is commonly assumed in other populations). Thus each “new” detection cannot be assumed to reflect the incidence of a new episode of AOM. There were 24.4 and 22.2 child-years of intervention for amoxicillin and placebo groups, respectively. In the placebo group, we detected one quarter of an episode of normal ears per child per year (one in 4 years), 2.8 episodes of AOM without perforation, and 2.2 episodes of AOM with perforation per child per year. Bilaterally normal ears were detected in the amoxicillin group more than 3 times as often as in placebo children. Continuous amoxicillin more than halved the incidence (detection) of AOM with perforation. (Table S3a) The incidence (detection) of nasopharyngeal carriage with each bacterial pathogen was between 8 and 10 per child year in the placebo group. Amoxicillin reduced the incidence (detection) of pneumococcal infection from 9.1 to 6.9 episodes per child per year. (Table S3b)

| **Table S3a: Incidence per child year and incidence rate ratio (IRR) of the following worst ear status during therapy** | | | | |
| --- | --- | --- | --- | --- |
|  | **Amoxicillin**  **24.4**  **child years** | **Placebo**  **22.2**  **child years** | **IRR**  **[95% CI]**  **Adjusted for child** | **p** |
| 1. Normal | 0.74 | 0.23 | 3.2 [1.2, 9.0] | 0.025 |
| 1. AOM without perforation | 3.3 | 2.8 | 1.2 [0.78, 1.7] | 0.469 |
| 1. AOM with perforation | 0.95 | 2.2 | 0.45 [0.23, 0.87] | 0.018 |

| **Table S3b: Incidence per child year and incidence rate ratio (IRR) of nasopharyngeal carriage of the following OM pathogens**^¥^ **during therapy** | | | | |
| --- | --- | --- | --- | --- |
|  | **Amoxicillin**  **24.4 child years** | **Placebo**  **22.2 child years** | **IRR**  **[95% CI]**  **Adjusted for child** | **p** |
| 1. Spn | 6.9 | 9.1 | 0.76 [0.66, 0.86] | 0.000 |
| 1. NCHi | 8.3 | 8.1 | 1.0 [0.89, 1.2] | 0.779 |
| 1. M. cat | 9.9 | 10.4 | 0.95 [0.88, 1.0] | 0.188 |
| 1. All Spn, NCHi and M.cat | 5.4 | 6.8 | 0.79 [0.66, 0.95] | 0.014 |
| 1. Penicillin intermediate or high level resistant Spn (MIC > 0.1μg/ml) | 3.9 | 4.6 | 0.84 [0.62, 1.2] | 0.283 |
| 1. Beta-lactamase producing NCHi | 0.6 | 0.12 | 1.9 [0.99, 3.7] | 0.052 |

^¥^ Spn *Streptococcus pneumoniae*. NCHi non-capsular *Haemophilus influenzae*. M.cat *Moraxella catarrhalis.*
